# Effects of Low Pressure Treatment on the Mortality of Different Life Stages of Indianmeal moth (*Plodia interpunctella*) and the Quality of Dried Chinese Jujube

**DOI:** 10.3390/insects9040136

**Published:** 2018-10-06

**Authors:** Juncai Hou, Lushuai Zhao, Shaojin Wang

**Affiliations:** 1 College of Mechanical and Electronic Engineering, Northwest A&F University, Yangling, Shaanxi 712100, China; houjuncai@nwsuaf.edu.cn (J.H.); zhaolushuai135@163.com (L.Z.); 2 Department of Biological Systems Engineering, Washington State University, 213 L.J. Smith Hall, Pullman, WA 99164-6120, USA

**Keywords:** mortality, Indianmeal moth, life stage, jujube, quality, low pressure

## Abstract

Low pressure technology is a potential non-chemical method to control insects in agriculture products. The purpose of this study was to determine the tolerance of different life stages of indianmeal moth (*Plodia interpunctella*) to low pressure and to validate the mortality of *P. interpunctella* when infesting Chinese jujube (*Zizyphus jujuba* Miller) using a low pressure system. Results showed that larvae were the most tolerant life stage of *P. interpunctella*, with a minimum exposure time of 41.4 h at 1.3 kPa and 25 °C to obtain 100% mortality. Pupae were the most susceptible life stage, with a lethal time of 12 h under the above low pressure conditions. The survival ratio of *P. interpunctella* in jujube decreased with increasing exposure time and reached 0% when the jujubes with *P. interpunctella* larvae were exposed to low-pressure treatment for 41.6 h at 1.3 kPa and 25 °C. Although the color, moisture, soluble solid, and vitamin C contents were slightly changed, there was no significant quality difference in these factors between control and treated jujubes. The information provided by this study is useful in developing effective non-chemical low-pressure treatments for disinfesting agricultural products.

## 1. Introduction

Chinese jujube (*Zizyphus jujuba* Miller) is indigenous to China with a history of over 4000 years [1]. The Chinese jujube is favored by people for its nutrients, especially vitamin C (V_c_), and can be stored for a long time after drying. Indianmeal moth, *Plodia interpunctella* (Hübner) (Lepidoptera: Pyralidae), is one of the main pests of dried jujube during long storage [2] and one of the most serious insect pests in stored products worldwide [3]. It causes damage to an extremely large variety of dry stored products, such as beans, chocolate, dried fruits, grains, and nuts [4]. It is reported that these infestations in stored products can cause up to 9% economic loss in developed countries and 20% in developing countries [5,6]. At present, methyl bromide (MeBr) and phosphine fumigation are the most popular methods to control these stored insects because they can be applied easily with low cost, high effect, and broad spectrum [7]. However, chemical fumigation may leave harmful residues on stored products and cause negative effects on the environment and human health [8]. Furthermore, some stored insects may have enhanced insecticide resistance with increasing application times of fumigants [9,10,11]. Therefore, there is an urgent need to develop alternative physical methods to replace chemical fumigation for disinfesting jujubes.

One of the possible non-chemical alternative methods is the use of low-pressure technology to disinfest stored and fresh products, since it is environmentally sustainable and residue free [12,13,14,15]. The main mechanism of low-pressure insecticidal action is deemed to be hypoxia affecting cell physiological processes but not dehydration from low water concentration under low pressure [16]. Researchers used low pressure technology to kill eggs and larvae of the Caribbean fruit fly, *Anastrepha suspensa* (Diptera: Tephritidae), in mangoes at 1.9 kPa, ≥ 98% RH, and 13 °C [13]. Adult life stages of the almond moth, *Ephestia cautella* (WIK.) (Lepidoptera, Phycitidae), were shortened at all low pressures and all moths died within 14 h at 13.3 kPa [17]. The mortality of 5th-instar codling moth, *Cydia pomonella* (L.) (Lepidoptera: Tortricidae) larvae, which were the most tolerant life stage, reached 98% after 12 days of exposure to 10 °C and 1.33 kPa with almost saturated humidity, and the acceptable quality of the ‘Red Delicious’ apples was maintained [14]. It has been reported that insect mortality during low pressure pretreatments increases with increasing temperature [18,19,20], and different life stages respond differently to various low-pressure treatment conditions [20]. For example, the lethal time for the lesser grain borer, *Rhyzopertha dominica* (Fabricius), was 278 h at 5 °C and 50 mm Hg, but it reduced sharply to 79 h when the temperature increased to 30 °C [21]. Therefore, it is important to determine the most tolerant life stage and the lethal conditions for *P. interpunctella* in jujube when developing an effective low-pressure treatment protocol.

After the lethal conditions for stored insects in low pressure or vacuum treatments are determined, it is also necessary to analyze the treatment effect on the product quality. The product quality of apple, iceberg lettuce, and local seeds after low pressure treatments is retained well at low temperatures [14,22,23]. It is desirable to determine the effects of low-pressure treatments on jujube quality at ambient temperature.

The main objectives of this study were: (1) to investigate the tolerance of *P. interpunctella* at different life stages to low pressure treatment, (2) to validate the practical low pressure treatment protocol using jujube infested with the most tolerant life stage, under different exposure times at 25 °C with 1.3 kPa, and (3) to evaluate the quality of dried jujube before and after low pressure treatment.

## 2. Materials and Methods

### 2.1. Low Pressure System

**Figure 1:**
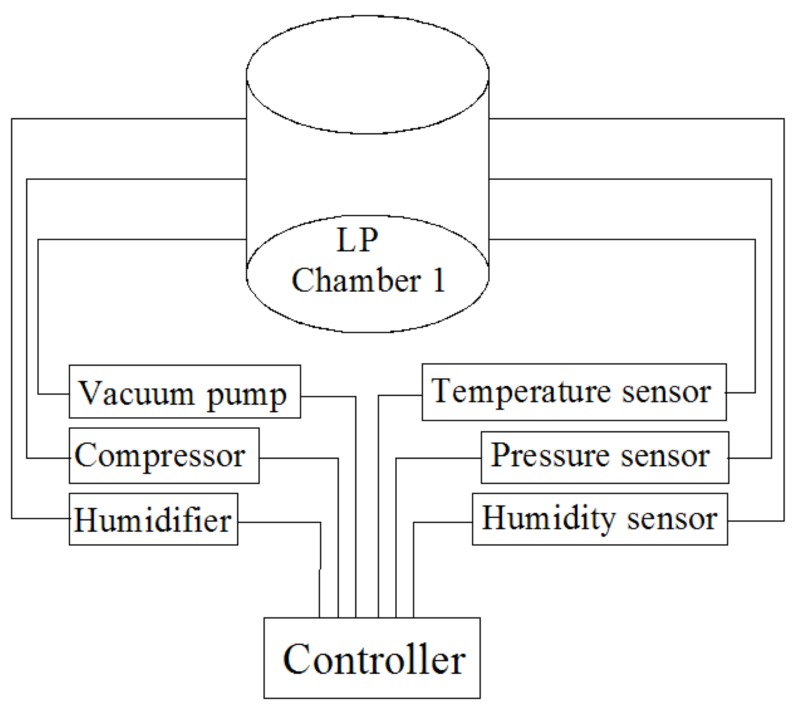
Diagram of the low pressure (LP) system, including the vacuum pump, compressor, and humidifier.

A low-pressure system (DC-0.15, Dachang refrigeration equipment engineering Co. Ltd., Jiangxi, China), consisting of 5 identical stainless steel low pressure chambers (37 cm diameter × 30 cm height), was used in this study. The setting pressure, humidity, and temperature of each chamber were obtained by a vacuum pump, humidifier, and compressor, and these parameters were independently measured and recorded in each chamber during the treatment with the accuracy of ± 200 Pa, ± 2 °C, and ± 2% RH, respectively. All data from the five humidity, pressure, and temperature sensors in the low-pressure system were sent to the same controller and displayed on a same screen. A diagram of one of the identical low-pressure chambers is shown in Figure 1.

### 2.2. Chinese Jujubes

Chinese jujubes were bought from Younike Cooperation, Zhengzhou, Henan Province, China. The average individual weight and moisture content of the jujubes were 2.13 ± 0.31 g and 20.69 ± 2.16% (w.b.), respectively. Plastic bags containing 500 g of jujubes were kept at ambient room temperature (25 ± 1 °C) until use. The shelf life of the jujubes provided by the manufacturer was nine months.

### 2.3. Test Insects

*Plodia interpunctella* were reared for several generations at the Academy of State Administration of Grain, Beijing, China, and obtained in July 2017. Larvae of *P. interpunctella* were fed on mixed food with 35% unbleached wheat flour, 35% corn flour, 7% oats, 7% honey, 7% yeast, 7% glycerol, and 2% soybean flour by weight. About 20 g of mixed feed and suitable larva were placed into glass bottles (8 cm diameter × 10 cm height) sealed by filter papers for air exchange and maintained at 28 ± 1 °C and 60 ± 5% RH in a constant temperature and humidity chamber (HWS-350, Hangzhou Aipu Instrument Co. Ltd., Hangzhou, China), with a photoperiod of 14:10 (L:D) h with artificial light. Adults were put into glass bottles (20 per bottle) covered with gauze with a mesh size of 1 mm. The glass bottles were then inverted on the bottom of glass petri dishes with 9 cm diameter for collecting eggs. One-day eggs were collected and used for the low pressure treatment. Fifth-instars of *P. interpunctella* were collected when they crept from the rearing media to the bottle wall or cover and put into a new glass bottle for future use. The pupae of *P. interpunctella* were collected when their color had changed to light brown. Since adult *P. interpunctella* have already demonstrated susceptibility at 4.3 kPa [20], eggs, larvae, and pupae were chosen for determining their tolerance to low pressure.

### 2.4. Treatment Procedures

Based on pilot experiments, five exposure times (5–25 h with 5 h intervals for eggs, 8–40 h with 8 h intervals for larvae, and 2–12 h for pupae) at 1.3 kPa, 25 °C, and 60 ± 5% RH were chosen to span survival ratios from 100% to 0% and to determine the most low-pressure-tolerant life stage of *P. interpunctella*. The control eggs, larvae, and pupae were placed into the low-pressure chamber at 101.325 kPa, which is equivalent to ambient atmospheric pressure at sea level, 25 °C, and 60 ± 5% RH, and kept for 25, 40, and 12 h, respectively. *Plodia interpunctella* eggs were put directly on the bottom of glass petri dishes. One dish containing 100 eggs was used for each treatment. About 50 larvae or pupae, placed in glass bottles and covered with filter paper, were used for each treatment including the controls. After petri dishes or glass bottles with treated insects were put into the low-pressure chamber, the treatment time was counted from when the chamber pressure reached 1.3 kPa. Because of leaks, the pressure had a slight rise. Once the pressure reached 1.5 kPa, the LP system worked again until it remained at 1.3 kPa. After the low-pressure treatment, treated insects were kept under the rearing conditions for mortality observation.

Chinese jujube was infested with fifth-instar larvae of *P. interpunctella,* which was the most low-pressure-tolerant stage of *P. interpunctella*. Each of the 30 infested jujubes per treatment was infested with a larva, which was then allowed to penetrate the jujube overnight under the rearing conditions. Each of the 30 infested jujubes was placed into a glass bottle sealed by filter paper and exposed to low pressure treatment for 8, 16, 24, 32, or 40 h at 1.3 kPa, 25 °C, and 60 ± 5% RH. Control infested fruits were placed into the low-pressure chamber at 101.325 kPa, 25 °C, and 65% RH and incubated for 40 h. After the low pressure and control treatments, the infested jujubes were moved to rearing conditions until evaluation.

The petri dishes with treated eggs were kept for at least 10 days after the treatment for counting hatched and unhatched eggs. Mortality of eggs was estimated as the percentage of unhatched eggs relative to total treated eggs. Larvae mortality was evaluated for 24 h after treatment. Larvae were considered to be dead if their body was dark or if they did not respond to a light probe. Missing larvae in infested jujubes were considered as dead. Treated and control pupae were held for 6–10 days until adult emergence. All moving adults were considered as surviving of pupae. Larval and pupal mortality were calculated as the dead larvae or pupae relative to total treated numbers. The mortality for *P. interpunctella* eggs, larvae, and pupae were corrected based on control mortality using Equation (1) proposed by Abbott [24]:

(1) $$Mortality_{cr}=\frac{Mortality_{o}(\%)-Mortality_{c}(\%)}{100-Mortality_{c}(\%)}\times 100\%\;$$

where *Mortality_cr_* means the corrected mortality of insects, *Mortality_o_* is the observed mortality of treated insects, and *Mortality_c_* stands for the mortality of controls.

### 2.5. Chinese Jujube Quality Analyses

The quality of control samples and Chinese jujubes treated under 1.3 kPa, 25 °C, and 60 ± 5% RH for 40 h was evaluated. Moisture content, color, soluble solid content, and vitamin C content were selected as major parameters to evaluate Chinese jujube quality.

The moisture content of jujubes was measured with a moisture analyzer (HE53, Mettler-Toledo, Shanghai, China). The soluble solid content (SSC; %) of the sample was determined with a digital Abbe refractometer (DR-A1, Atago China Guangzhou Corporation Ltd., Guangzhou, China). Vitamin C content was measured with 2,6-dicholrindophenol titration by following the method reported by others [25].

Jujube color was determined with a computer vision system (CVS). Detailed information on the CVS and the operation of color image processing was described by Hou et al. [26]. Color images of 5 jujubes from each treatment were taken by the camera and stored in a laptop. The *L*, *a*, and *b* values of jujubes were obtained by Adobe Photoshop CS (Adobe Systems Inc., San Jose, CA, USA) and converted to Commission International Eclairage (CIE) LAB (L*, a*, and b*) values using the following equations [24,27]:

(2) $$L^{*}=\frac{L}{2.5}\;$$

(3) $$a^{*}=\frac{240}{255}a-120\;$$

(4) $$b^{*}=\frac{240}{255}b-120\;$$

### 2.6. Statistical Analysis

Each low-pressure treatment, including controls, was repeated three times. The average values and standard deviations were computed from the three replicates. The mortality ratio for treated insects was corrected using the Abbot formula. All statistical analyses were carried out at a 5% significance level using the statistical software SPSS version 16.0 (SPSS Inc., Chicago, IL, USA).

## 3. Results and Discussion

### 3.1. Survival of Different Life Stages at Low Pressure

**Figure 2:**
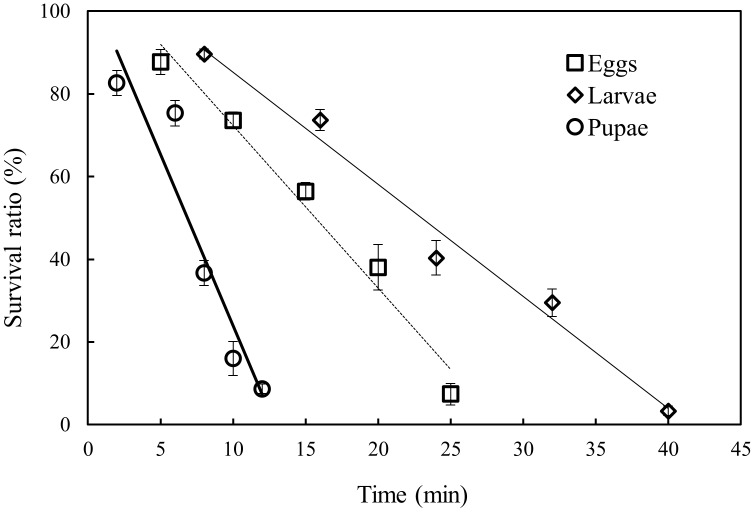
The survival ratios for *P. interpunctella* eggs, larvae, and pupae under different exposure times at 1.33 kPa and 25 °C.

The control survival ratios for *P. interpunctella* eggs, larvae, and pupae were 54.33, 98.25, and 93.71%, respectively. The low survival ratio for *P. interpunctella* eggs could have been caused by cannibalism [28] or handling processes. The survival ratios, corrected using Equation (1), for different life stages of *P. interpunctella* under different exposure times at 1.33 kPa and 25 °C are shown in Figure 2. The survival ratio of all developmental stages of *P. interpunctella* decreased with increasing exposure time, and 100% mortality of eggs, larvae, and pupae was obtained at the minimum exposure times of 28.4, 41.4 and 12.9 h, respectively. However, the survival ratio of *P. interpunctella* pupae decreased slightly at the beginning of low pressure treatment and then sharply after that period (Figure 2). The insecticidal mechanism of low pressure treatment is the effects of reduced oxygen levels in combination with relative humidity and temperature. With increasing exposure time, the oxygen was consumed by the treated insects and the survival ratio of treated insects decreased. This phenomenon was found for cowpea weevil, *Callosbruchus maculates* (F.) (Coleoptera: Bruchidae) [19], codling moth, *Cydia pomonella* (L.) (Lepidoptera: Torticidae) [14], and nondiapausing and diapausing *P. interpunctella* larvae [18].

Lethal time (LT_50_ and LT_95_) results from the probit analysis for different life stages of *P. interpunctella* and exposure times under low pressure treatment are listed in Table 1. Larvae of *P. interpunctella* were the life stage most tolerant to low pressure, and 40.12 h were required at 1.3 kPa and 25 °C to achieve 95% mortality. The pupae were the life stage most susceptible to low pressure, and 12.18 h were needed at 1.3 kPa and 25 °C to achieve 95% mortality. Differences in tolerance among life stages can also be observed from the slopes of probit lines (Table 1). The larvae had the lowest slope value, while the slope value of pupae was the highest, indicating that the shortest exposure time was needed for pupae to achieve 100% mortality. These results agreed with those found for *Cydia pomonella* [14] and *P. interpunctella* [29]. However, different results were found for *Callosbruchus maculatus* [19], in which the larvae were the most susceptible life stage. This could have been caused by the different pressure and size of low pressure chamber. Generally, active larvae, that are presumed to have higher oxygen demand than egg and pupae, were found to be the most susceptible life stage in smaller low-pressure chambers. As they were the most tolerant life stage, the larvae were chosen as the target life stage for validation studies.

### 3.2. Protocol Validation Studies with Infested Jujube

Treatment results showed that the insect survival ratio for the control was 100%, indicating that the handling procedure did not influence the survival ratio of *P. interpunctella* larvae. The survival ratio of *P. interpunctella* larvae decreased from 86.99% to 0% when the exposure time increased from 8 h to 40 h at 1.3 kPa and 25 °C. In general, increasing exposure time at given pressures resulted in decreased survival ratios. The survival ratio curve for *P. interpunctella* larvae was described by the linear regression equation *S* = −2.55*t* + 106.16, with the coefficient of determination *R^2^* = 0.97, where *t* is exposure time (h) and *S* is the survival ratio (%) of *P. interpunctella* larvae. The minimum exposure time determined by this equation was 41.6 h to achieve 100% mortality. This result was close to the lethal time determined by directly placing *P. interpunctella* larvae in the low-pressure chamber, suggesting that the low-pressure treatment may have good diffusion and high penetration into the jujube samples [30].

### 3.3. Jujube Quality

Table 2 summarizes the major quality parameters of control and treated jujubes. The results showed that jujube quality was not significantly affected (*p* > 0.05), even though the moisture content, SSC, Vc, and color were slightly changed. Since the moisture content of treated jujubes was lower than humidity of chamber, the treated jujubes might absorb some water from the chamber during long exposure times, resulting in the moisture content of treated jujubes being slightly higher than that of the controls. The a* value of treated jujubes was slightly higher than that of the controls, indicating that the control jujubes were less red than treated samples. Although the low-pressure treatment may prolong the shelf life of most fruits, such as apples [14], studies on jujube sensory quality during long storage are needed to develop the practical low-pressure treatment protocol.

## 4. Conclusions

Lethal times of eggs, larvae, and pupae to obtain 100% mortality at 1.3 kPa and 25 °C were 28.9, 41.4, 12.9 h, respectively. Larvae were the most tolerant life stage and pupae were the most susceptible life stage under the above low-pressure conditions. An exposure of 41.6 h at 1.3 kPa and 25 °C can completely control all larvae of *P. interpunctella* infested in jujubes. Although the moisture content, color, Vc, and SSC were slightly changed after treatment, there were no significant differences between control and treated jujube quality. The current study showed that low pressure treatment has the potential to be an alternative non-chemical disinfestation method for jujubes.

## Figures and Tables

**Table 1:** Lethal time (hours) for *P. interpunctella* life stages exposure to 1.3 kPa at 25 °C.

Stage	*n*	Slope ± SE	*R^2^*	Min. Time for 100% Mortality	LT_50_	95% CI		LT_95_	95% CI	
						Lower	Upper		Lower	Upper
eggs	300	4.18 ± 0.14	0.972	28.4	15.28	14.37	17.38	26.87	22.57	31.18
larvae	150	2.79 ± 0.03	0.986	41.4	23.00	21.05	24.22	40.12	36.01	46.23
pupae	150	8.72 ± 0.38	0.889	12.9	7.10	6.30	8.56	12.18	9.19	15.17

**Table 2:** Quality characteristics (mean ± SD) of control and treated jujubes for 40 h, at 1.3 kPa and 25 °C.

Treatment	Moisture (% w.b)	SSC (%)	Vc (mg/100g)	Color		
				L*	a*	b*
control	20.69 ± 2.15a	76.52 ± 2.13a	232.81 ± 11.01a	29.78 ± 10.36a	27.36 ± 5.21a	21.22 ± 3.64a
treated	21.36 ± 1.85a	78.16 ± 2.51a	225.27 ± 10.73a	29.28 ± 9.83a	32.26 ± 5.37a	22.89 ± 4.03a

^a^ Same letters in a column indicate that means are not significantly different at *p* = 0.05 between control and treated jujubes.
